# Is Prosthetic Dentistry Advancing as It Should

**Published:** 1899-02

**Authors:** A. N. Davis

**Affiliations:** Piqua, O.


ARTICLE IV.

### IS PROSTHETIC DENTISTRY ADVANCING AS IT SHOULD.

A. N. DAVIS, PIQUA, O.

In writing this paper I sorrowly say, that I am an undergraduate and realize that my statements will not have the power of those from the pen of some well known man of the profession, whose statements would be based upon years of experience.

Observation, study and intercourse with some of the best men in denistry, cause me to feel the needs, of which I write.

In most of the large cities in this country, few men know what it is to have laboratories, and those that have are generally very poorly supplied with all the modern appliances and instruments necessary for the best work; others have only a roll top desk as such, while three-fourths send their work to a mechanical dentist, of whose ability they know little and very seldom see the work until it is finished.

It is generally kept secret from patients that their work is done by a dentist other than the reputable man whose ability and skill they trusted, and I am sure we, ourselves, would not care to have work in our own mouths made hastily, and with little thought and less ability.

The hard work of Drs. Bonwill, Burchard, and Molyneaux, does not seem to be appreciated by the majority, and the Bonwill articulator is more of an ornament, or used only as a straight line articulator, very few understanding its principle and movements in articulation.

In the *Dental Review* of May, '98, there is a paper, the subject of which is, "Prophylaxis in Bridge Work," the writer showing the necessity of such, even in the best bridge skill can produce.

It is almost an impossibility to thoroughly cleanse all the surfaces of the natural lower molars, and many of the others. Then how much more difficult to keep a bridge clean with its undercuts and irregular spaces?

The aforesaid writer advises the patient to return at the end of six months, to have the bridge thoroughly cleansed with pumice, silk floss, etc.; that it is impossible for him to keep it in a clean condition himself. If a well made and artistic bridge, "which is accomplished by few," will become filthy in six months, it will become filthy in a week, and it would be well to advise the patient to return and have the bridge cleaned three times a day.

It will be surprising to a great many to know that Dr. Bonwill never makes a bridge and never puts on a gold crown, always using his "Bonwill" crown or the English tube tooth made by C. Ash & Son.

When one or more teeth are absent, posterior to the first bicuspid, and enough left to which to clasp, the Bonwill Removable Plate Bridge given and illustrated in "Evans' Artificial Crown and Bridge Work," page 264, is without doubt the best appliance that can be used.

In case one or two of the incisors are missing, where the removable plate bridge cannot be used on account of clasping, a properly constructed small gold bridge might be used to advantage, as in such a position it could be kept perfectly clean and occlusion could be made perfect.

The saddle back porcelain bridge invented by E. Parmly Brown of New York, given in the "American Text-Book of Prosthetic Dentistry," is the ideal for replacing artificial teeth, but as yet, it has many disadvantages, which, perhaps, will be overcome by time and experience in that class of work.

The profession needs more investigation and study in this work, and as man's ability in operative work may be judged by skill and art displayed in prosthetic work.

I think these statements will be indorsed by a man who has done more for prosthetic dentistry than any man in the world—Dr. W. G. A. Bonwill.—*Items of Interest.*

